# Editorial: Insights in precision medicine: 2021

**DOI:** 10.3389/fmed.2023.1145876

**Published:** 2023-03-03

**Authors:** Alice P. Chen

**Affiliations:** AP Chen Consultant, Potomac, MD, United States

**Keywords:** precision medicine, genomics, education, hyperthyroidism, technology

Precision medicine has been a hot topic in recent years as I have the privilege of seeing these changes in all fields especially in oncology. It offers the potential to revolutionize healthcare by tailoring treatment to a specific target. This approach, which considers genetic, environmental, and lifestyle factors, as well as more recently metabolomic and proteomic approaches has the potential to improve patient outcomes and reduce the risk of negative side effects. The recent Research Topic, “*Insights in precision medicine: 2021*,” shed light into the diverse research even on the eve of COVID-19 and provide future challenges in the field of precision medicine. The nine articles published showcase the growth of the area and the increasing unanswered questions that each discovery brings. These articles are wide range from using AI to implement treatment to education and covers cancer to chronic conditions such as epilepsy and diabetes.

One key area of focus in precision medicine is the use of genomic information to guide treatment decisions. This is particularly relevant in the context of diseases such as renal cell carcinoma, hepatocellular carcinoma, and liver diseases, where specific genomic factors can be used to predict patient outcomes and guide treatment choices.

Four articles highlight the potential use of precision medicine in improving patient outcomes for chronic disorder outside of oncology. “*Long non-coding RNA MALAT1: A key player in liver diseases*” by Lu et al. discuss the multiple roles of MALAT1 which as often in nature can be beneficial or harmful. It is beneficial when regeneration of liver is needed but overexpression could lead to proliferation and metastasis. “*Low-dose everolimus maintenance therapy for renal angiomyolipoma associated with tuberous sclerosis complex*” by Luo et al. albeit a small study showed the long-term treatment of everolimus in renal angiomyolipoma. “*EEG-driven prediction model of oxcarbazepine treatment outcomes in patients with newly-diagnosed focal epilepsy*” by Wang et al. presents a prediction model based on electroencephalography data that is able to accurately predict treatment outcomes in patients with focal epilepsy being treated with oxcarbazepine. “*Genome-wide association study of hyperthyroidism based on electronic medical record from Taiwan*” by Liu et al. identifies genetic risk factors for hyperthyroidism, which can be used to guide treatment.

Schaibley et al.'s and Field's articles highlight the challenges and opportunities involved in implementing genomics-based precision medicine in clinical practice. “*Limited genomics training among physicians remains a barrier to genomics-based implementation of precision medicine*” raise the awareness that genomics training for healthcare professionals needs to start at the medical school level to teach physicians how to use genomic data in patient care. “*Bioinformatic challenges detecting genetic variation in precision medicine programs*” discusses the challenges involved in using bioinformatics techniques to analyze and interpret genomic data in precision medicine programs, including the need to develop robust data management systems and to address issues of data privacy and security. The use of artificial intelligence (AI) in training and use for precision medicine can help to address these challenges by providing tools and algorithms that can automate the analysis of genomic data and support the development of personalized treatment plans. However, it is important to ensure that these AI-based approaches are validated and that they are used in a responsible manner that considers the unique needs and circumstances of individual patients.

The rest of the articles emphasize precision medicine in oncology; all highlight the potential of precision medicine in improving patient outcomes and tailoring treatment to the specific needs of individual patients in oncology. “*A somatic mutation signature predicts the best overall response to anti-programmed cell death protein-1 treatment in epidermal growth factor receptor/anaplastic lymphoma kinase-negative non-squamous non-small cell lung cancer*” by Peng et al. demonstrates the potential of using a somatic mutation signature, a profile of genetic changes present in a tumor, to predict the best overall response to anti-PD-1 treatment in non-small cell lung cancer. “*Precision medicine: An optimal approach to patient care in renal cell carcinoma*” by Sharma et al. discusses the genomic changes that are associated with renal cell carcinoma and how these changes can be used to guide treatment decisions. “*Development and validation of a prognostic signature associated with tumor microenvironment based on autophagy-related lncRNA analysis in hepatocellular carcinoma*” by Deng et al. presents a prognostic signature based on the analysis of long non-coding RNAs associated with the tumor microenvironment that is able to accurately predict patient outcomes in hepatocellular carcinoma. These studies show that precision medicine has the potential to revolutionize the management of cancer by providing personalized treatment strategies based on the unique characteristics of each patient's disease.

In conclusion, precision medicine is changing the way medicine is implemented and continues to improve patient outcomes. As significant as the advances are in this field, there are still challenges to be addressed including education of physicians in this area and new technologies to drive the science toward precision, the benefits of precision medicine are clear, and it is essential that we continue to invest in and support research in this field.

## Author contributions

The author confirms being the sole contributor of this work and has approved it for publication.

